# High-efficiency super-elastic liquid metal based triboelectric fibers and textiles

**DOI:** 10.1038/s41467-020-17345-8

**Published:** 2020-07-15

**Authors:** Chaoqun Dong, Andreas Leber, Tapajyoti Das Gupta, Rajasundar Chandran, Marco Volpi, Yunpeng Qu, Tung Nguyen-Dang, Nicola Bartolomei, Wei Yan, Fabien Sorin

**Affiliations:** Institute of Materials, Ecole Polytechnique Fédérale de Lausanne (EPFL), Lausanne, 1015 Switzerland

**Keywords:** Devices for energy harvesting, Electronic devices, Polymers

## Abstract

Fibers that harvest mechanical energy via the triboelectric effect are excellent candidates as power sources for wearable electronics and functional textiles. Thus far however, their fabrication remains complex, and exhibited performances are below the state-of-the-art of 2D planar configurations, making them impractical. Here, we demonstrate the scalable fabrication of micro-structured stretchable triboelectric fibers with efficiencies on par with planar systems. We use the thermal drawing process to fabricate advanced elastomer fibers that combine a micro-textured surface with the integration of several liquid metal electrodes. Such fibers exhibit high electrical outputs regardless of repeated large deformations, and can sustain strains up to 560%. They can also be woven into deformable machine-washable textiles with high electrical outputs up to 490 V, 175 nC. In addition to energy harvesting, we demonstrate self-powered breathing monitoring and gesture sensing capabilities, making this triboelectric fiber platform an exciting avenue for multi-functional wearable systems and smart textiles.

## Abstract (web summary)

Current triboelectric fibers suffer from complex fabrication schemes and relatively poor performance. Here the authors use a highly scalable thermal drawing process to make soft and resilient micro-structured triboelectric fibers and textiles with applications both as sensors and energy harvesters.

## Introduction

Flexible electronics and particularly soft fibers and functional textiles are becoming an ideal platform in the areas of biomedical and health monitoring, implants and prosthesis, motion tracking, artificial intelligence, and human-machine interaction^1–4^. A key challenge for their sustainable operation lies in the necessity of reliable and low-profile power systems. This is particularly important for implanted or textile-integrated systems, to untether them from current heavy and rigid batteries that require frequent charging or replacement^5–7^. One promising strategy to alleviate this challenge is the utilization of energy-harvesting technologies to sustainably generate electricity from the surroundings. Examples include solar cells to capture energy from sunlight^8^ and thermoelectric generators to produce electricity from temperature gradients^9,10^. Another promising strategy recently developed lies in triboelectric nanogenerators (TENGs) that combine contact-electrification and electrostatic induction effects to generate electricity from various mechanical stimuli, such as friction, vibration, rotation, and expanding/contracting motions^11–13^. Owing to a wide choice of materials and promising electrical outputs, various TENG configurations have been developed to adapt to different application scenarios^14,15^.

Thus far, however, the performance and properties of TENG configurations within fibers and textiles remain limited and unpractical. Fibers and fabrics obtained from coatings or printing remain complex to implement and exhibit performance that is below state-of-the-art planar configurations^16,17^. Another strategy for smart textiles and wearable technologies involves integrating functionality directly at the fiber level^18–21^. To be practical and efficient, however, the fiber devices must host complex micrometer-scale architectures, be soft, and sustain various, sometimes large, deformation to generate a high-voltage output over several cycles. The fibers must also be thin so that they can deform under low forces, to ensure maximum energy-harvesting output, as well as better sensing performance. Furthermore, the fabrication approach must be scalable and versatile enough to comply with different post-processing treatments, including the integration into textiles.

To date, commonly-used materials, such as polymer coatings^22^ and metallic wires^23^, are rigid, and even when engineered in more deformable configurations, such as wavy and coiled architectures^24^, the performance still remains limited. Other configurations, such as thick, coaxial^25–27^, twisted^28^, and interlaced^29^ cable-like TENGs that could incorporate multiple insulative and conductive materials, have been proposed. To further enhance softness, liquid metal was also introduced within bulky silicon rubber constructs to make single-electrode TENGs^30^. While more stretchable, these devices remain of large feature size, and exhibit limited output performance, especially with respect to volume. Moreover, bulky systems are not well adapted for deformation sensing as they require higher forces to be activated. Their fabrication also involves several complicated steps that are typically restricted to laboratory scale, resulting in short-length and large-diameter cables that are not compatible with a variety of other applications, such as implants, e-skin, textile weaving, and knitting^23,26,30,31^. The production, at an industrially relevant scalability, of thin and stretchable fiber- and textile-based TENGs with advanced architectures allowing for comparable performance with conventional planar devices, still presents great challenges from the perspectives of fiber materials, designs, and processing technologies.

Herein, we report the design and scalable fabrication of liquid metal-based microstructured stretchable TENG fibers and textiles with performance comparable with state-of-the-art planar configurations. Specifically, we identify a thermoplastic elastomer that combines better triboelectric performance than commonly used materials, with rheological attributes compatible with the highly versatile fiber thermal drawing technique. Thanks to these findings, we demonstrate the fabrication of potentially kilometer-long microstructured TENG fibers with advanced cross-sectional architectures integrating multiple liquid metal electrodes and a micrometer-scale surface texture. The resulting fibers are highly deformable, being able to sustain complex deformations and extreme elongations up to 560%. Thanks to the combination of thin elastomeric cladding with an extra surface area brought upon by the engineered texture, and deformable yet conductive liquid metal electrodes, the fibers can efficiently harvest energy from mechanical friction/vibration stimulation. Moreover, thermally drawn triboelectric fibers are thin and flexible enough to be seamlessly woven into an elastic and machine-washable energy-harvesting textiles. As a result, we could fabricate a 36-cm^2^ textile that exhibits an open-circuit voltage (*V*_oc_) and a short-circuit transferred charge (*Q*_sc_) as high as 490 V and 175 nC, respectively, which equals state-of-the-art performance of two-dimensional planar TENGs with similar dimensions. Thanks to their specific design and high deformability, fibers can also form highly efficient and self-powered embedded deformation sensors capable of monitoring low-force stimuli such as breathing. The fiber cladding we identified is also highly resilient, and the fiber devices maintain high outputs even after repeated extreme deformations and extended environmental exposure. The advanced fiber design and processing approach we propose brings fiber and textile design flexibility, fabrication scalability, and state-of-the-art performance to the field of fiber-based triboelectric nanogenerators. As a result, soft thermally drawn microstructured fibers can address the challenges associated with the integration of low- profile and efficient powering units within implants, wearable devices, and advanced textiles.

## Results and discussion

### Design and fabrication of superelastic triboelectric fibers

**Fig. 1:**
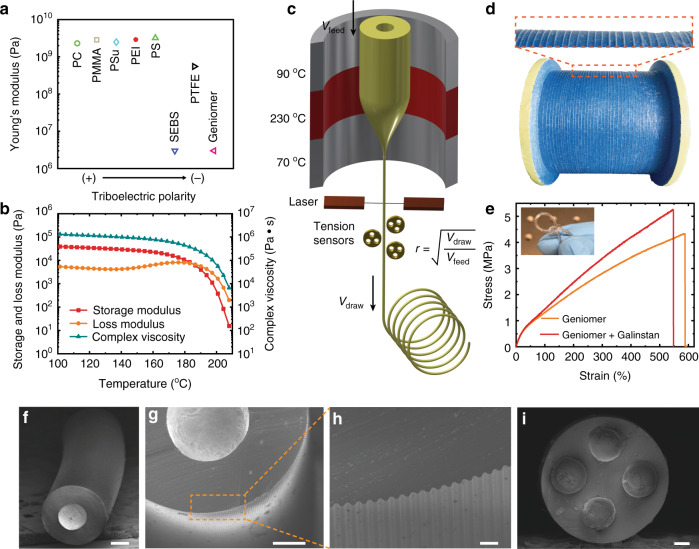
Materials characterization and fiber fabrication. **a** Comparison of Young’s modulus and relative triboelectric polarity of electrical insulating polymers that are compatible with the thermal drawing process. PTFE is also listed for a better comparison. **b** Rheological properties of Geniomer: storage modulus (G’), loss modulus (G”), and complex viscosity. **c** Schematic showing thermal drawing process for the production of long fibers. **d** A roll of 40 m of continuous fiber obtained from a single draw, demonstrating the scalability of drawing process. **e** Stress–strain curves of a fiber without and with an embedded liquid metal electrode. The inset shows a fiber being randomly knotted. **f** SEM image of a fiber with a single liquid metal electrode. Scale bar: 200 μm. **g**, **h** SEM image of a fiber with microtextured surface. Scale bar: 200 μm (**g**) and 20 μm (**h**). **i** SEM image showing a fiber with four liquid metal electrodes. Scale bar: 200 μm. Note the abbreviations of the polymers in **a**: PC polycarbonate, PMMA poly(methyl methacrylate), PSu polysulfone, PEI polyetherimide, PS polystyrene, SEBS poly(styrene-*b*-(ethylene-*co*-butylene)-*b*-styrene), PTFE polytetrafluoroethene.

The working principle of a TENG system with a single electrode surrounded by an insulating cladding is based on the combination of contact-electrification and electrostatic-induction effects^32^. The contact-electrification effect occurs between the microstructured fiber and surrounding materials. From a triboelectric potential table of various elements (Supplementary Fig. 1)^33,34^, we can observe that the most commonly used materials in our everyday life tend to positively charge when contact electrification takes place. To ensure a high electrical output from soft fibers, we must select fiber materials that are compatible with the thermal drawing process, exhibit elastomer-like mechanical properties at room temperature, and possess a negative potential upon electrification. In Fig. 1a, we show the Young’s modulus (*E*) and relative triboelectric polarity of a few materials compatible with the thermal drawing process. We also show for reference the data for polytetrafluoroethene (PTFE), a material with a strongly negative triboelectric polarity. Among these polymers, we selected Geniomer, a two-phase block copolymer made up of a soft polydimethylsiloxane phase and a hard aliphatic isocyanate phase. Geniomer is a thermoplastic elastomer with interesting mechanical deformation properties and especially a low Young’s modulus (*E* = 3.4 MPa), combined with a higher negative triboelectric polarity compared with PTFE. Geniomer also possesses the proper rheological properties to be compatible with the thermal drawing process, with a viscous flow-like behavior at high temperature^35,36^, as shown in Fig. 1b. In addition to the cladding material, another key constituent for stretchable triboelectric fibers is a compatible electrode material with both high electrical conductivity and good stretchability. We oriented our choice toward a liquid metal alloy formed by gallium, indium, and tin (Ga62/In22/Sn16, named Galinstan), which exhibits not only high deformability but also a high electrical conductivity (3.46 × 10^6^ S m^−1^) typical of metallic materials^37,38^.

After identifying the proper materials, we schematically show the working principle of the thermal drawing process in Fig. 1c, which was initially developed to fabricate optical fibers. The process starts with the fabrication of a macroscopic version of the targeted fiber, termed preform, which is machined to the desired architecture (see details in “Methods” and Supplementary Fig. 2)^39,40^. Subsequently, the preform is locally heated above the glass transition temperature of the cladding (here Geniomer) in a furnace and continuously pulled into a uniform and long fiber^41,42^. The dimensions of the fibers, monitored in real time by a laser system, are controllable by tuning the furnace temperature distribution, the preform feeding velocity, and the fiber drawing speed^43^. With this approach, potentially hundreds of kilometers of fibers with the same structure as the preform can be reliably fabricated (Fig. 1d). Two strategies could be adopted for the integration of the liquid metal. For the sufficiently large channels and shorter fiber lengths (typically tens of meters), a straightforward strategy consists of infiltrating the metal post-drawing within the fiber microchannels. For small-diameter channels, the liquid metal is integrated in the proper channels at the preform level, where it flows as a liquid within the polymer structure. Our experiments showed that the electrical resistance of the liquid metal increases with an elevated temperature (Supplementary Fig. 3), but can recover to its original value after cooling down.

Figure 1e and Supplementary Fig. 4 show the recorded stress–strain curves of a fiber without and with one liquid metal channel. The two fiber designs display almost identical elastomeric features with fracture strain reaching up to 599 ± 73% and 557 ± 54%, and Young’s modulus as low as 3.4 ± 0.6 MPa and 3.8 ± 0.3 MPa, respectively. A photograph showing a triboelectric fiber under high deformation is presented in Supplementary Fig. 5. In the inset of Fig. 1e, we show that the triboelectric fibers are capable of tolerating other types of sophisticated deformations such as tight knots. In addition, the liquid metal can maintain its high electrical conductivity even under large strain, as we show in Supplementary Fig. 6.

In Fig. 1f and Supplementary Fig. 7, we show the cross-sectional structure of fibers with one integrated liquid metal electrode. The versatility of the thermal drawing process can be further exploited to realize more complex fiber architectures with enhanced performance. It has been demonstrated in particular that TENG systems with rough surfaces can exhibit enhanced output^44^. In Fig. 1g, h, we show the cross section of a fiber with a textured surface that exhibits enhanced performance compared with smooth fibers, as we show below. The design of the periodic square-shaped patterns on the preform was chosen because of the ease of fabrication, and the ability of such structures to be maintained and avoid excessive reflow during thermal drawing^19,45,46^. In Fig. 1i, we also demonstrate a fiber with four integrated liquid metal-based electrodes that can also exhibit enhanced performance, as we analyze below.

### Electrical output characterization of triboelectric fibers

We next turn to investigate the ability of soft thermally drawn fibers to work as TENG devices. We start by characterizing a simple fiber configuration with one liquid metal within a Geniomer cladding, and will demonstrate the effect of adding more complex features such as a textured surface or multiple channels toward the end of the paper. In Supplementary Fig. 8, we schematically show the working principle of soft fibers triggered by repeated contact-separation movements with a poly(methyl methacrylate) (PMMA) sheet. It is assumed that, in the original state (before mechanical excitation), there are no electric charges on the fiber and PMMA surfaces, and the liquid metal electrode is connected to the ground. As an external force is applied to the PMMA sheet, it is brought into contact with the fiber surface, resulting in positive charges on the surface of the PMMA sheet and negative charges on the fiber surface, respectively. As the PMMA sheet moves back away from the fiber, positive charges are removed from the surrounding of the metallic electrode that is felt as a change in the electrostatic field. Similarly, when the charged PMMA sheet comes back into contact, an inverse field change is induced. An alternating current flowing from the liquid metal into the ground is hence generated at each cycle. Similarly, taking into account the mechanical deformation of the fiber, the same process also takes place at the interface between the fiber and the bottom PMMA, although with less inductive electron flow. To understand the electricity-generating process more quantitatively, the electric potential distribution of the system was simulated via finite-element analysis using COMSOL. The 3D model was established based on the real structure and dimensions of the fiber, while material properties were assigned in the software. We assumed that the whole fiber structure was exposed to air, and the potential at infinity was set to zero. The two tribo-charged surfaces are assigned with charge density of ±10 μC m^−2^, respectively.

**Fig. 2:**
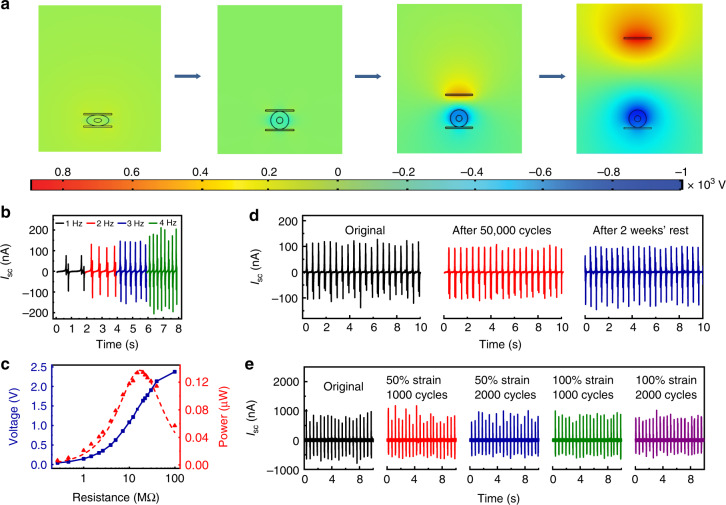
Electrical output performance of triboelectric fibers. **a** Finite element simulation of the potential distribution of a fiber with a single liquid metal electrode under open-circuit condition. **b** Variation of I_sc_ with frequency of stimulation (contact length: 3 cm). **c** Dependence of the output voltage and peak instantaneous power on the loaded resistors. **d** Long-term output stability tests under continuous pressing for 50,000 cycles, together with the outputs after being stored in ambient atmosphere for 2 weeks (contact length: 3 cm). **e** Variation of I_sc_ after repeated stretching cycles at 50 and 100% strain for 2,000 cycles, respectively. A longer fiber with the contact length of 10 cm and a slightly larger pressure were used.

The electric potential was calculated using the electrostatics module, assuming that the surface charges can be kept on the surfaces for an extended time due to their insulative nature. Figure 2a shows the cross-sectional view of the model with simulated electrical potential distribution when the PMMA sheet is released and moving further away with varied distance. It is observed that with the movement of the PMMA sheet, the triboelectric charges are separated and induce a significant change to the electrostatic field, consistent with the proposed mechanism in Supplementary Fig. 8.

To quantitatively assess the fibers’ energy-harvesting performance, we characterized their electrical outputs triggered by periodic vertical compression of a PMMA sheet, driven by a DC motor (Supplementary Fig. 9). In Fig. 2b and Supplementary Fig. 10, we show typical outputs of a short fiber (contact length: 3 cm) under various frequencies. While there is no obvious change for *V*_oc_ (~5.5 V, 269.9 V/cm^3^) and *Q*_sc_ (~1.6 nC, 78.5 nC/cm^3^), the peak value of short-circuit current (*I*_sc_) reveals an increasing trend with the frequency, from ~90 nA (4.4 µA/cm^3^) at 1 Hz to ~180 nA (8.8 µA/cm^3^) at 4 Hz. This is ascribed to the enhanced flow rate of charges induced by higher deformation rates of the fiber at higher frequencies^47^. Compared with other previously reported triboelectric fiber constructs (Supplementary Table 1), thermally drawn fibers with advanced cross-sectional architectures represent a significant improvement in terms of not only fabrication process and textile integration, but also in terms of output performance. The output power of the fiber was investigated by externally connecting various resistors from 0.3 MΩ to 100 MΩ in series, and calculated as $\frac{{V^2}}{R}$, where *R* is the load resistance and *V* is the voltage across it. As shown in Fig. 2c, the instantaneous output power achieves a peak value at a moderate *R* value. Since the principle of TENGs is based on electrostatic induction and has inherent capacitive behavior, the output power *P* could also be expressed as

1 $$P = \frac{{V_{oc}^2R}}{{|Z_c|^2 + R^2}},$$

where *V*_oc_ is the open-circuit voltage of the fiber nanogenerator, |*Z*_*c*_| is the magnitude of its internal impedance^48^. By fitting the output power values with this formula (indicated by a dash line in Fig. 2c), we can find that the maximum output power is located at the load resistance of 17.4 MΩ. Thus, according to the load-match condition for maximizing the output power on the load, the magnitude of the fiber’s internal impedance is estimated to be 17.4 MΩ.

To quantify the impact of repetitive deformation on the fiber’s energy-harvesting performance, we repeatedly compressed and relaxed the fiber at 2 Hz, while simultaneously monitoring the short-circuit current waveforms (Fig. 2d). The magnitude of the current after 50,000 cycles remains 96% of its initial value. In addition, the reproducible output results after 2 weeks’ rest further confirm its high reliability. The fibers were then subjected to repeated large elongation deformation. As shown in Fig. 2e, the current from the fiber remains stable after being stretched to 50 and 100% strain values for 2,000 cycles. Therefore, we confirm that the fiber is capable of withstanding cyclic external compression and tensile forces while maintaining excellent energy-harvesting performance for a long period of time. This can be attributed to the resilient mechanical properties of the elastomer and excellent electrical conductivities of the liquid metal under large deformation.

### Stretchable triboelectric textiles for energy harvesting

**Fig. 3:**
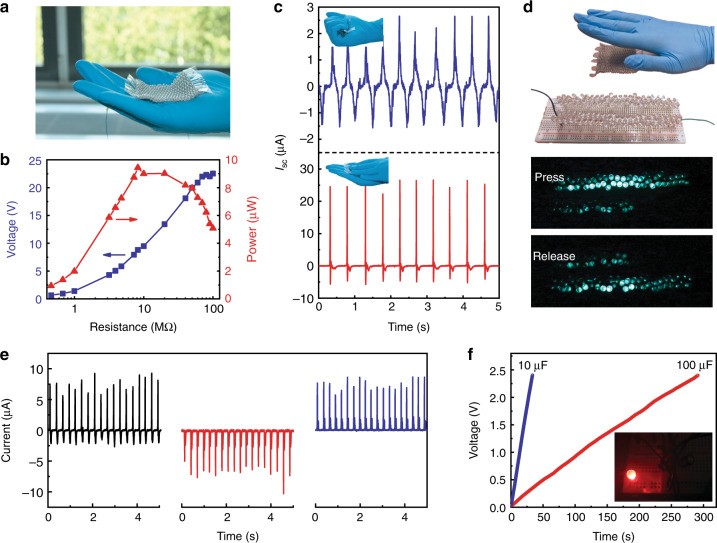
Energy-harvesting performance of triboelectric textiles. **a** Photograph showing that a continuous and long triboelectric fiber was woven into a textile. **b** Dependence of the output signals on the resistance of external loads. **c** *I*_sc_ waveforms of the textile triggered by mechanical stimuli: hand grasping (upper part) and hand tapping (bottom part). **d** Demonstration of 100 LEDs lighted up by the textile under tapping. The connection of the LED arrays is schematically shown in Supplementary Fig. 12. **e** The output current of the textile before and after being rectified by a bridge rectifier. **f** Charging curves of two different capacitors connected to the textile. The inset shows that a red LED can be continuously lighted up by a charged capacitor.

Having characterized the fibers’ mechanical and electrical output performance, we now turn to study their potential to be integrated into two-dimensional textiles for sustainable and self-powered applications. A conformal and stretchable textile (6 cm × 6 cm) was obtained by simply weaving a single continuous 4-m-long and 1.1-mm-thick triboelectric fiber, as shown in Fig. 3a. The effective output parameters of the textile on various resistive loads show the same trends as with the short fiber, while the instantaneous power achieves its peak value at a lower resistance of about 8.2 MΩ (Fig. 3b). To demonstrate the textile’s ability to take advantage of human motions for energy harvesting, we monitored its short-circuit current waveforms while being mechanically stimulated. For instance, hand grasping of the textile can generate high values of *V*_oc_, *I*_sc_, and *Q*_sc_, up to ~260 V, 2.5 μA, and 85 nC, respectively (Fig. 3c; Supplementary Fig. 11). If a stronger stimulation like hand tapping is applied, the corresponding parameters can achieve as high as ~317 V, 26 μA, and 130 nC, respectively (Fig. 3c; Supplementary Fig. 12). Such levels of outputs are sufficient to power 100 light-emitting diodes (LEDs) that are connected in reversed direction (Fig. 3d; Supplementary Fig. 13, Supplementary Movie 1).

Since TENGs produce pulsed and alternating output that cannot be exploited by most electronic devices, a rectifier circuit is necessary. Supplementary Fig. 14 shows the circuit diagram of a bridge rectifier to modulate outputs of the textile to charge a capacitor. As shown in Fig. 3e, the alternating current can be effectively converted into positive or negative pulsating current as desired. Figure 3f shows the voltage versus time curves of two different commercial capacitors (10 μF and 100 μF) that are charged by the rectified current generated by hand-tapping a functional textile. The electric energy stored in the capacitors could be further employed to continuously drive a LED (inset in Fig. 3f), which confirms the capability of the textile to harvest sufficient mechanical energy from human daily motion to drive some portable electronic devices without any external power sources.

**Fig. 4:**
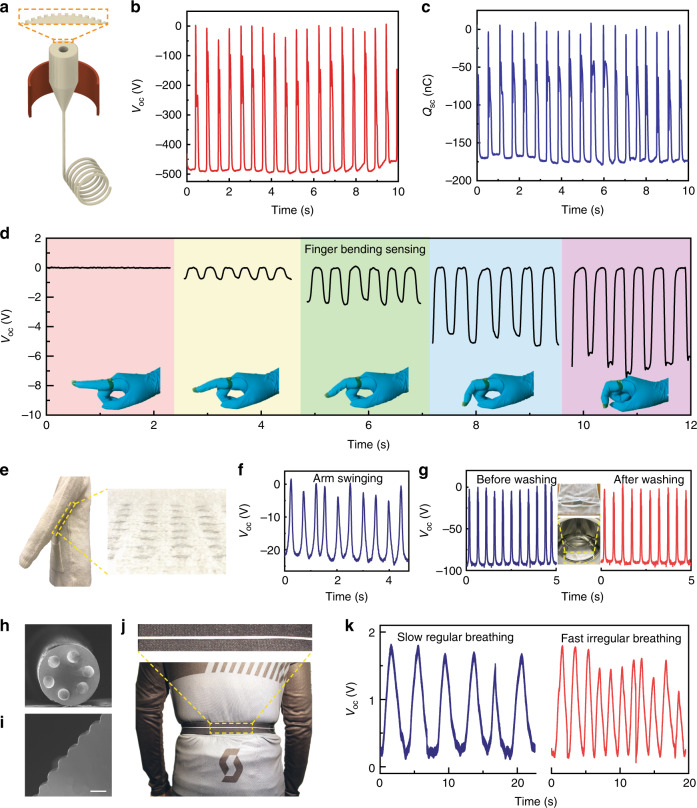
Triboelectric fibers and textiles for wearable applications. **a** Schematic of the thermal drawing of a microtextured soft fiber. **b**, **c** The recorded *V*_oc_ and *Q*_sc_ of a 6 cm × 6 cm textile made up of a long microtextured fiber, under the applied force of 25.5 N (7.1 kPa). **d** Demonstration of the fibers as self-powered sensors to monitor finger-bending conditions. **e**, **f** Photographs and the corresponding *V*_oc_ of a triboelectric fiber-integrated sweater to extract energy from human arm swing. **g** Comparison of the voltage output of the textile before and after being washed in a laundry machine. The inset image shows a water-permeable protective sack with the textile placed in a household laundry machine. **h**–**j** Breathing monitoring with a self-powered triboelectric wearable sensor. The fiber features six embedded electrodes (**h**) and a microtextured surface (**i**) for improved performance. The fiber is fixed on a stretchable belt, which is worn around the torso (**j**). **k** The generated waveforms allow the quantitative assessment of breathing patterns. Scale bars: **h** 200 μm, **i** 10 μm.

The flexibility associated with thermal drawing can be further exploited to fabricate triboelectric fibers with complex architectures for enhanced efficiencies. We demonstrate in particular fibers with multiple liquid metal-based electrodes or microtextured surfaces shown in Fig. 1g–i, both of which show enhanced output performance, as shown in Supplementary Figs. 15, 16. To demonstrate the impact of such advanced microstructuring, the electric output of a triboelectric textile (6 cm × 6 cm) formed by a microtextured fiber was characterized, as shown in Fig. 4a–c. The peak values of *V*_oc_ and *Q*_sc_ triggered by hand tapping are as high as ~490 V (13.6 V/cm^2^) and ~175 nC (4.86 nC/cm^2^), which are estimated to be 55 and 42% higher than the texture-free counterpart, respectively. These values match the calculated surface area increase from a flat to a texture surface, which can indeed host a higher charge (see Supplementary Note 1). In Supplementary Table 2, we summarized the used materials, dimensions, and electrical outputs, and calculated electrical output densities (calibrated into unit area) of various textiles reported in recent years. From such a list, we conclude that the performance of our textile surpasses reported TENG textiles, and is comparable with, or even superior to, most 2D planar TENG configurations reported thus far.

### Wearable applications of multi-functional fibers and textiles

As we have shown, triboelectric fibers can generate electrical signals when triggered by an external force. The collected signals can be utilized to extract characteristics of the mechanical stimuli^49,50^, assigning the fiber a second function—self-powered sensor. Here, a finger-gesture sensor was built by simply attaching the triboelectric fiber onto a glove, and was characterized by recording its output when the index finger is bent. As shown in Fig. 4d, the amplitude of the *V*_oc_ waveforms increases gradually with the increasing bending angle, indicating an opportunity of the soft fiber as a wearable self-powered mechanical sensor. In particular, this fiber design shows interesting advantages, compared with many other fiber-shaped mechanical sensors that suffer from the problems of low sensitivity, limited sensing range, or structural and functional damage when exposed to large deformations^51^.

A peculiarity of the thin and soft fibers lies in their possibility to be integrated within textiles using a variety of well-established techniques such as knitting and weaving. As shown in Fig. 4e, we integrated soft triboelectric fibers into commercial clothes for the construction of functional clothing. Driven by the friction from a swinging arm between the fiber and the sweater, an output voltage of ~22 V was produced (Fig. 4f). For wearable electronic products, the endurance to day-to-day handling, like machine washing, is also one of the most critical factors determining their viability in practical settings^29^. The electrical outputs of the textile before and after washing in a laundry machine for 40 min are almost identical (Fig. 4g), indicating its good tolerance to harsh conditions, such as water infiltration and the impact force induced by the machine. Such performance can be attributed to the high deformability of the fiber together with the thick elastomeric cladding that protects the fiber inner structure.

Finally, as shown in Fig. 4h, i, we propose a novel fiber design with a surface texture and up to six liquid metal electrodes, to realize a highly sensitive self-powered deformation sensor to monitor breathing. To demonstrate the potential application of such a fiber, we built a breathing monitoring belt by fixing the fiber extremities to the two ends of a longer stretchable belt. Tightly worn around the torso as shown in Fig. 4j, the outward and inward movements of the upper chest and abdomen result in the elongation/release of both the belt and the fiber, inducing friction between the two due to their different intrinsic deformation behavior. This slight relative movement generates an electrical signal that can be detected, enabling the monitoring of breathing patterns as shown in Fig. 4k.

In this work, we demonstrated a simple and scalable fabrication approach of microstructured highly stretchable TENG fibers with architectures of unprecedented complexity and performance on par with state-of-the-art TENG devices. The fibers consist of a thermoplastic elastomeric cladding that surrounds one or multiple liquid metal electrodes, co-processed via the multimaterial thermal drawing technique. The produced fibers exhibit high stretchability with a fracture strain as high as 560%. We showed that the fibers could maintain stable performance even after 50,000 cyclic compression and long-term large deformation operations. We further optimized the energy-harvesting performance by integrating multiple electrodes and introducing textured pattern to the elastomer surface, resulting in a triboelectric textile with *V*_oc_ and *Q*_sc_ reaching as high as 490 V and 175 nC, respectively. The fabrication process we developed is scalable and enables kilometer-long fibers to be yielded from a single preform, leading to promising possibilities for large-area, flexible and soft sensing and energy-harvesting systems. For example, preforms in the optical fiber industry can produce 10^3^ km of 0.5-mm-thick TENG fibers in a single draw. Integrated via various textile-based technologies, a 500-m^2^ fabric could be fabricated that could generate a current as high as 3.5 A, enough to work as reliable power sources for smartphone batteries. Another advantage of our strategy is the high flexibility of the design of complex fibers at the preform level, such as textured surfaces or multiple electrodes (Supplementary Fig. 17). The compatibility with textile-integration techniques also opens novel avenues for advanced designs and self-powered functionalities within implants, wearable systems, and smart textiles. The self-powered and highly sensitive breathing monitoring application we demonstrated is being developed for the detection of early signs of breathing difficulties relevant for a variety of medical conditions in sleep pathologies or severe viral symptoms. We envision that this work not only has a significant impact in the fields of energy harvesting and functional fiber processing, but also provides opportunities for the advancement of other functional fiber and textile-based electronics.

## Methods

### Polymer comparison

The commonly used insulative thermoplastic polymers for thermal drawing were compared using the metrics of Young’s modulus and relative triboelectric polarity. The value of Young’s modulus for Geniomer was experimentally determined from mechanical tensile tests, while the other polymers were directly taken from corresponding data sheets or from literature^36^. The triboelectric polarity of the materials was determined from the electrical outputs of a series of self-constructed contact-mode 2D TENG devices with the selected polymer films as the dielectric surfaces (Supplementary Fig. 18).

### Fabrication of triboelectric fibers

The hollow-core cylindrical preforms were fabricated by consolidating Geniomer granules (140, Wacker Chemie AG) within molds at 175 °C in a vacuum-consolidation oven. The length and diameter of the preform are 120 mm and 29 mm, respectively. The microtextured Geniomer preforms were created by rolling a thin layer of Geniomer film with predefined texture onto the above-mentioned cylindrical preforms, followed by consolidation at 170 °C for 15 min under vacuum. The fabrication of the microtextured Geniomer film is as follows. Micropatterns with the defined period of 100 μm were created on silicon masks via photolithography process at the Centre for Micro-Nanofabrication (CMi) at EPFL. The patterns were made by a Heidelberg DWL200 laser writer on Cr-blank masks, then transferred to silicon masks by a Suss MA6 mask aligner. Afterward, the as-developed silicon masks were etched by a plasma etcher (Alcatel AMS 200 SE) to obtain the expected depth of 100 μm, which was designed to be the same as the width of the patterns, to get square-shaped patterns. The patterns were subsequently transferred to PDMS substrates by molding the silicon masks onto PDMS solution via casting (Sylgard 184 Dow-Coring) and curing at 80 °C. Geniomer thin films were prepared by hot pressing polymer granules under vacuum at 175 °C using a custom-built hot press (Laboratoire Presse, LAUFFER GmbH & Co. KG). Then the films were patterned by pressing on the patterned PDMS molds under vacuum at 170 °C.

Finally, the preforms were drawn into long fibers with a custom-built draw tower. The setting temperatures for top, middle, and bottom zones were 90 °C, 230 °C, and 70 °C, respectively. The preform was fed into the furnace at a speed of 1 mm/min, while the drawing speed was tuned with a wide range of 0.05–0.6 m/min to obtain fibers with controllable draw-down ratio.

### Characterizations

Scanning electron microscopy (SEM) was conducted on a Zeiss Merlin field-emission SEM with an acceleration voltage of 3 kV using the In-Lens detector and Analytic Column Mode. The fiber samples for SEM characterizations were prepared by immersing them into liquid nitrogen for several minutes followed by an immediate cutting at room temperature. All the samples were coated with a 10-nm Au film before characterization.

The rheological characterization of Geniomer was carried out on a TA Instruments AR 2000 Rheometer. The polymer plates (1500-μm thick) were cooled at 1 °C/min, and the angular frequency was 0.03 rad/s. The mechanical tensile tests of the fibers were conducted on a universal testing machine (UTS) Series LFM-125 kN (*Walter+Bai AG*) with the tensile mode (1-kN load cell, 2 mm/s). Five samples were characterized for each fiber type. The calculation of Young’s modulus from the stress–strain curves is limited to the low-strain region of less than 10% strain. The washing ability of the textile was characterized by putting the textile into a water-permeable protective sack, and the whole sack was put into a household laundry machine. The washing process lasted for about 40 min, after which the textile was dried in ambient condition.

### Electrical measurements

The electrical connection for the fibers was realized by contacting metallic wires to liquid metal electrodes followed by sealing with epoxy. The resistance of liquid metal electrodes was measured by an electrical testing instrument (Keithley 2450 Sourcemeter). A programmable electrometer (Keithley, model 6517) was utilized to measure the output signals of the triboelectric fibers and textiles. The data were collected and recorded by a Digilent Analog Discovery and computer-controlled software written in Labview. Custom setup composed of DC motors was used to apply cyclic pressing and stretching force to the tested fibers.

## Supplementary information

Supplementary Information

Description of Additional Supplementary Files

Supplementary Movie 1

## Data Availability

The authors declare that the data supporting the findings of this study are available within the article and the Supporting Information files. Raw data for the figures are available from the corresponding author upon reasonable request. Source data are provided with this paper.

## Source data

Source Data

## References

[1] Cherenack K, van Pieterson L (2012). Smart textiles: challenges and opportunities. J. Appl. Phys..

[2] Stoppa M, Chiolerio A (2014). Wearable electronics and smart textiles: a critical review. Sensors.

[3] Hu Y, Zheng Z (2019). Progress in textile-based triboelectric nanogenerators for smart fabrics. Nano Energy.

[4] Leber A, Cholst B, Sandt J, Vogel N, Kolle M (2018). Stretchable thermoplastic elastomer optical fibers for sensing of extreme deformations. Adv. Funct. Mater..

[5] Rogers JA, Someya T, Huang Y (2010). Materials and mechanics for stretchable electronics. Science.

[6] Lu N, Kim D-H (2014). Flexible and stretchable electronics paving the way for soft robotics. Soft Rob..

[7] Sun H, Zhang Y, Zhang J, Sun X, Peng H (2017). Energy harvesting and storage in 1D devices. Nat. Rev. Mater..

[8] O’Regan B, Grätzel M (1991). A low-cost, high-efficiency solar cell based on dye-sensitized colloidal TiO_2_ films. Nature.

[9] Bubnova O (2011). Optimization of the thermoelectric figure of merit in the conducting polymer poly(3,4-ethylenedioxythiophene). Nat. Mater..

[10] Zhang T (2017). High-performance, flexible, and ultralong crystalline thermoelectric fibers. Nano Energy.

[11] Luo J, Wang ZL (2019). Recent advances in triboelectric nanogenerator based self-charging power systems. Energy Storage Mater..

[12] Wang ZL, Chen J, Lin L (2015). Progress in triboelectric nanogenerators as a new energy technology and self-powered sensors. Energy Environ. Sci..

[13] Zhao J (2019). Remarkable merits of triboelectric nanogenerator than electromagnetic generator for harvesting small-amplitude mechanical energy. Nano Energy.

[14] Fan F-R, Tian Z-Q, Wang ZL (2012). Flexible triboelectric generator. Nano energy.

[15] Zhang Q (2019). Green hybrid power system based on triboelectric nanogenerator for wearable/portable electronics. Nano Energy.

[16] Zeng W (2014). Fiber-based wearable electronics: a review of materials, fabrication, devices, and applications. Adv. Mater..

[17] Huang Q, Wang D, Zheng Z (2016). Textile-based electrochemical energy storage devices. Adv. Energy Mater..

[18] Rein M (2018). Diode fibres for fabric-based optical communications. Nature.

[19] Nguyen-Dang T (2017). Multi-material micro-electromechanical fibers with bendable functional domains. J. Phys. D: Appl. Phys..

[20] Alexander Schmidt M, Argyros A, Sorin F (2016). Hybrid optical fibers—an innovative platform for in-fiber photonic devices. Adv. Opt. Mater..

[21] Stolyarov AM (2012). Fabrication and characterization of fibers with built-in liquid crystal channels and electrodes for transverse incident-light modulation. Appl. Phys. Lett..

[22] Yu X (2017). A coaxial triboelectric nanogenerator fiber for energy harvesting and sensing under deformation. J. Mater. Chem. A.

[23] Dong K (2017). 3D orthogonal woven triboelectric nanogenerator for effective biomechanical energy harvesting and as self‐powered active motion sensors. Adv. Mater..

[24] He X (2017). A highly stretchable fiber‐based triboelectric nanogenerator for self‐powered wearable electronics. Adv. Funct. Mater..

[25] Li X (2014). 3D fiber-based hybrid nanogenerator for energy harvesting and as a self-powered pressure sensor. ACS Nano.

[26] Dong K (2017). A highly stretchable and washable all-yarn-based self-charging knitting power textile composed of fiber triboelectric nanogenerators and supercapacitors. ACS Nano.

[27] Ryu J (2018). Intrinsically stretchable multi-functional fiber with energy harvesting and strain sensing capability. Nano Energy.

[28] Zhong J (2014). Fiber-based generator for wearable electronics and mobile medication. ACS Nano.

[29] Zhao Z (2016). Machine-washable textile triboelectric nanogenerators for effective human respiratory monitoring through loom weaving of metallic yarns. Adv. Mater..

[30] Yang Y (2018). Liquid-metal-based super-stretchable and structure-designable triboelectric nanogenerator for wearable electronics. ACS Nano.

[31] Lai Y-C (2017). Single-thread-based wearable and highly stretchable triboelectric nanogenerators and their applications in cloth-based self-powered human-interactive and biomedical sensing. Adv. Funct. Mater..

[32] Zhu G (2012). Triboelectric-generator-driven pulse electrodeposition for micropatterning. Nano Lett..

[33] Diaz AF, Felix-Navarro RM (2004). A semi-quantitative tribo-electric series for polymeric materials: the influence of chemical structure and properties. J. Electrostat..

[34] Wang ZL (2013). Triboelectric nanogenerators as new energy technology for self-powered systems and as active mechanical and chemical sensors. ACS Nano.

[35] Sordo F (2019). Microstructured fibers for the production of food. Adv. Mater..

[36] Qu Y (2018). Superelastic multimaterial electronic and photonic fibers and devices via thermal drawing. Adv. Mater..

[37] Zhu S (2013). Ultrastretchable fibers with metallic conductivity using a liquid metal alloy core. Adv. Funct. Mater..

[38] Fassler A, Majidi C (2015). Liquid-phase metal inclusions for a conductive polymer composite. Adv. Mater..

[39] Yan W (2019). Advanced multimaterial electronic and optoelectronic fibers and textiles. Adv. Mater..

[40] Page AG, Bechert M, Gallaire F, Sorin F (2019). Unraveling radial dependency effects in fiber thermal drawing. Appl. Phys. Lett..

[41] Leber, A. et al. Soft and stretchable liquid metal transmission lines as distributed probes of multimodal deformations. *Nat. Electron.*10.1038/s41928-020-0415-y (2020).

[42] Bayindir M, Abouraddy AF, Sorin F, Joannopoulos JD, Fink Y (2004). Detectors. Opt. Photonics N..

[43] Dong C, Page AG, Yan W, Nguyen-Dang T, Sorin F (2019). Microstructured multimaterial fibers for microfluidic sensing. Adv. Mater. Technol..

[44] Seung W (2015). Nanopatterned textile-based wearable triboelectric nanogenerator. ACS Nano.

[45] Nguyen Dang, T., Richard, I., Goy, E., Sordo, F. & Sorin, F. Insights into the fabrication of sub-100 nm textured thermally drawn fibers. *J. Appl. Phys.* **125**, 175301 (2019).

[46] Nguyen-Dang T (2017). Controlled sub-micrometer hierarchical textures engineered in polymeric fibers and microchannels via thermal drawing. Adv. Funct. Mater..

[47] Zhong J (2013). Finger typing driven triboelectric nanogenerator and its use for instantaneously lighting up LEDs. Nano Energy.

[48] Yi F (2016). A highly shape-adaptive, stretchable design based on conductive liquid for energy harvesting and self-powered biomechanical monitoring. Sci. Adv..

[49] Kim KN (2015). Highly stretchable 2D fabrics for wearable triboelectric nanogenerator under harsh environments. ACS Nano.

[50] Sim HJ (2016). Stretchable triboelectric fiber for self-powered kinematic sensing textile. Sci. Rep..

[51] Amjadi M, Kyung KU, Park I, Sitti M (2016). Stretchable, skin-mountable, and wearable strain sensors and their potential applications: a review. Adv. Funct. Mater..

