# Supplementary material for: High-efficiency super-elastic liquid metal based triboelectric fibers and textiles
Source: Nat Commun. 2020 Jul 15;11:3537. doi: 10.1038/s41467-020-17345-8 (PMC7363815; doi:10.1038/s41467-020-17345-8)
Supplement: Supplementary file 1 — Supplementary Information [file 41467_2020_17345_MOESM1_ESM.pdf]

Supplementary Information

High-efficiency Super-elastic Liquid Metal based Triboelectric Fibers and Textiles

Dong *et al.*

Supplementary Figures

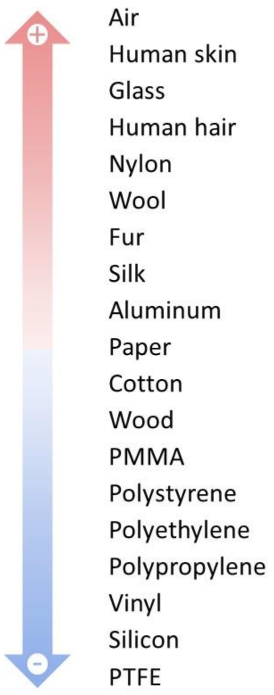

Supplementary Figure 1. Triboelectric series of various materials. "+" / "-" indicate whether a material tends to be positively or negatively charged when triboelectrification occurs.[1,2]

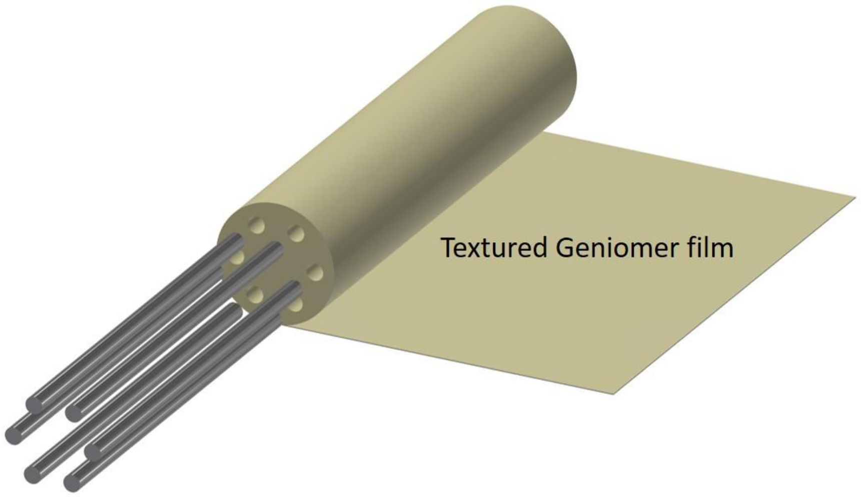

Supplementary Figure 2. Schematic of the preform fabrication. The preform integrates multiple channels and a microtextured surface.

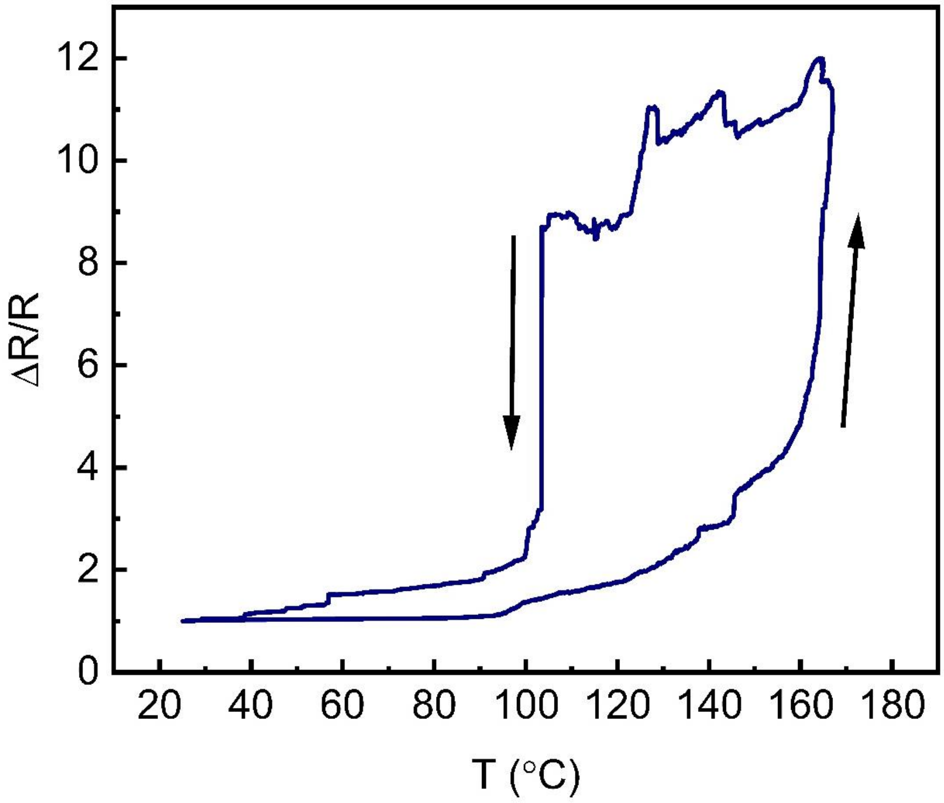

Supplementary Figure 3. Resistance change of Galinstan as a function of temperature. The tested temperature ranges from 25 to 165 °C (the highest temperature that Geniomer experienced during drawing process is around 150 °C).

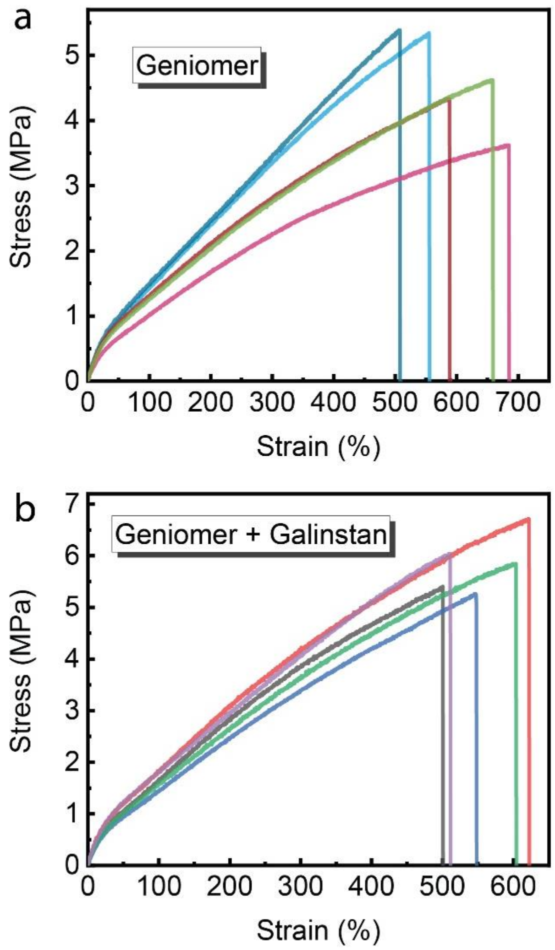

Supplementary Figure 4. Stress-strain curves of fibers. a Geniomer fibers without liquid metal. **b** Geniomer fibers with integrated liquid metal electrodes.

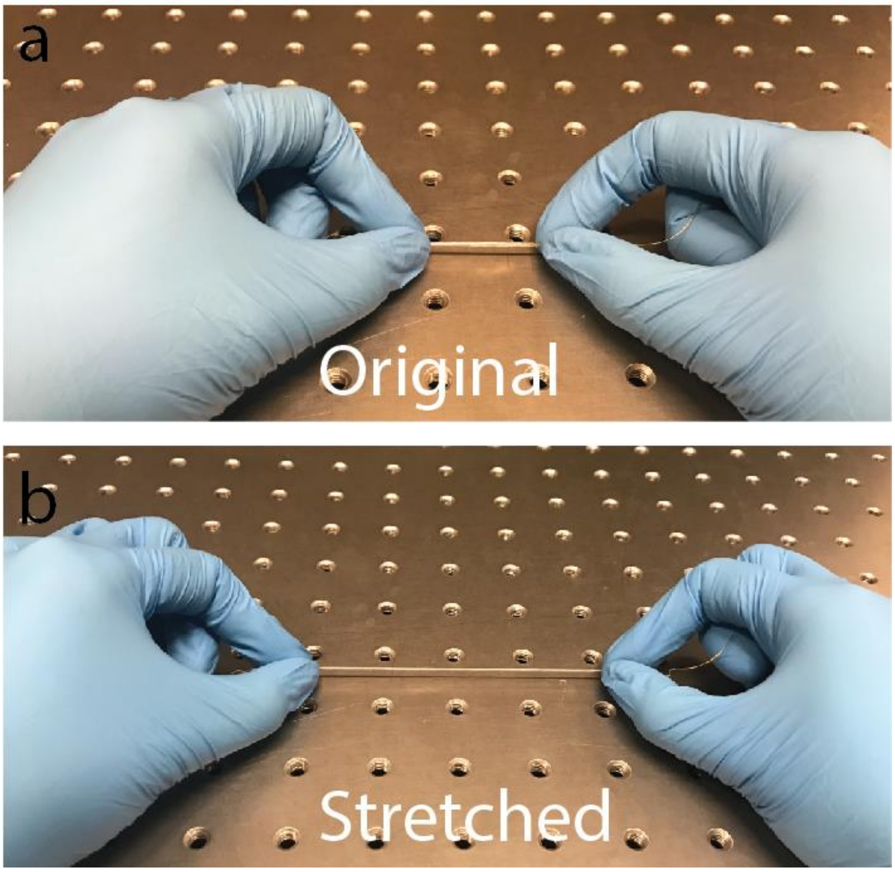

Supplementary Figure 5. Photographs showing the stretchability of triboelectric fibers. a A fiber with its original length. **b** The fiber being stretched to 400% strain.

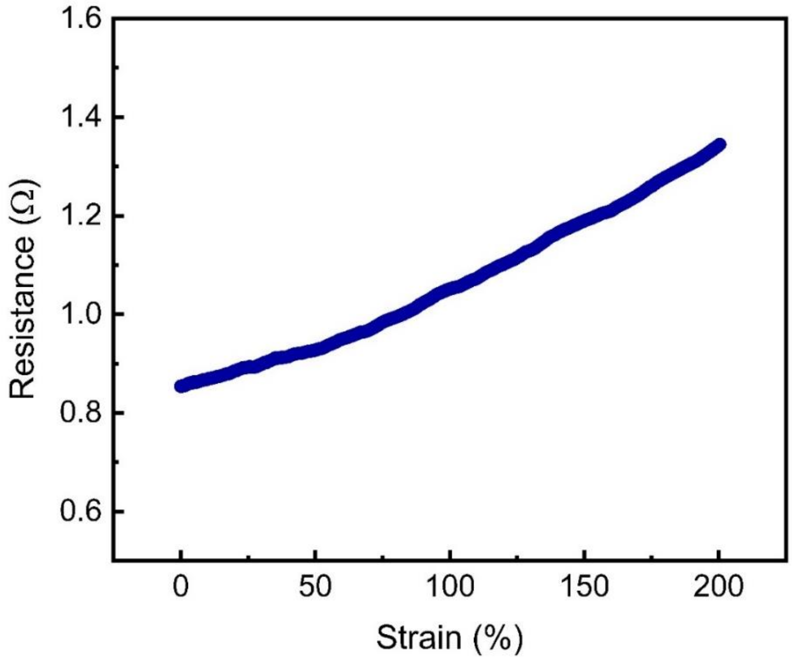

Supplementary Figure 6. Resistance change versus strain of the triboelectric fiber.

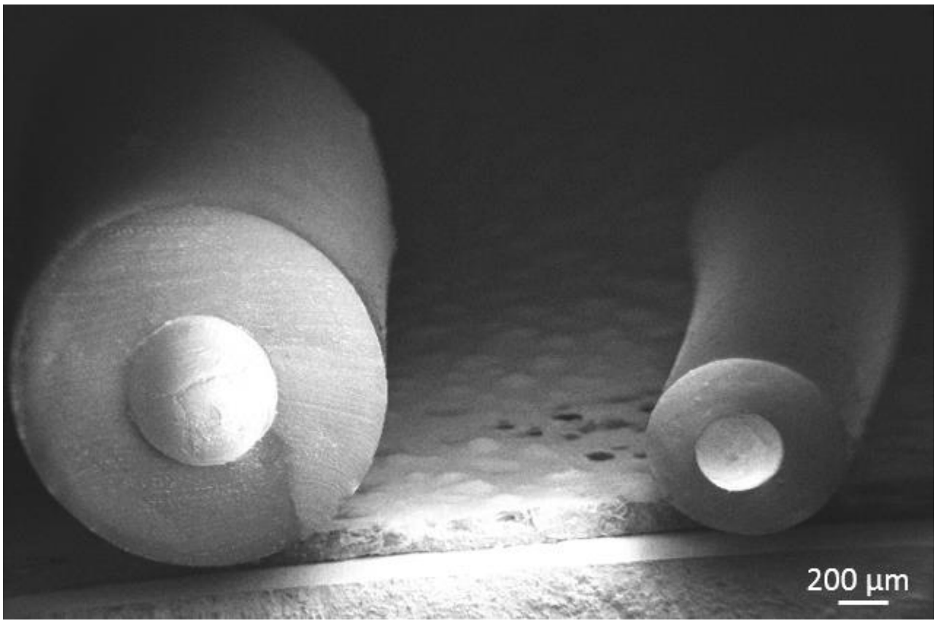

Supplementary Figure 7. SEM image showing the fibers with tunable dimensions.

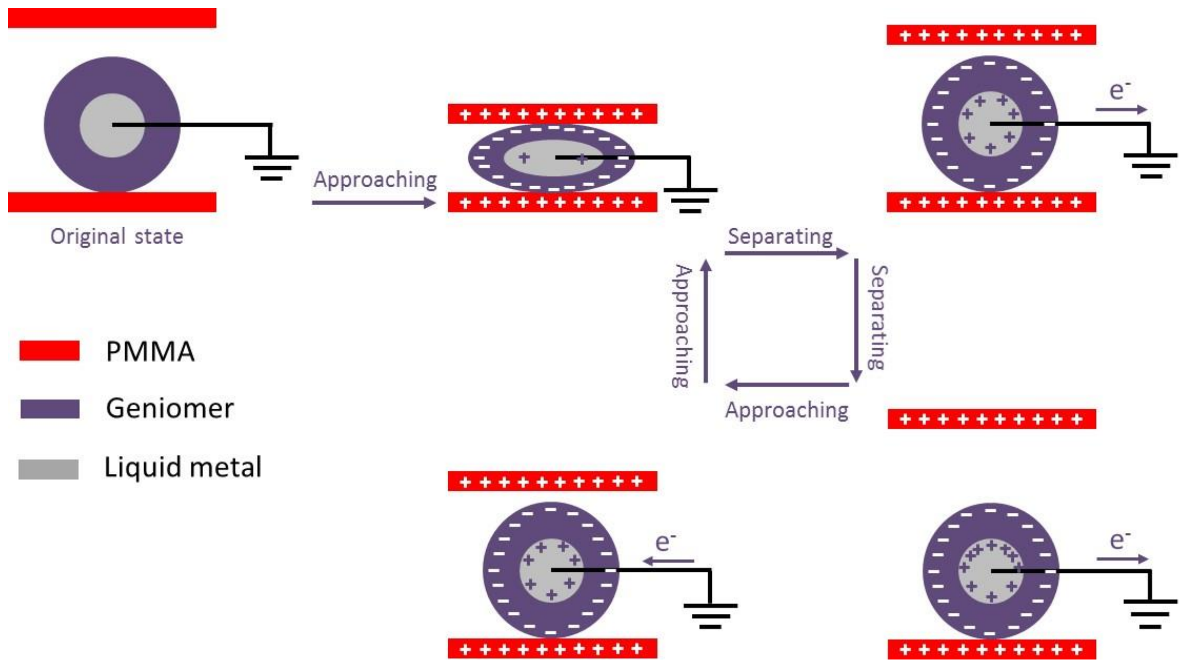

Supplementary Figure 8. Charge distribution of a TENG fiber under short-circuit conditions.

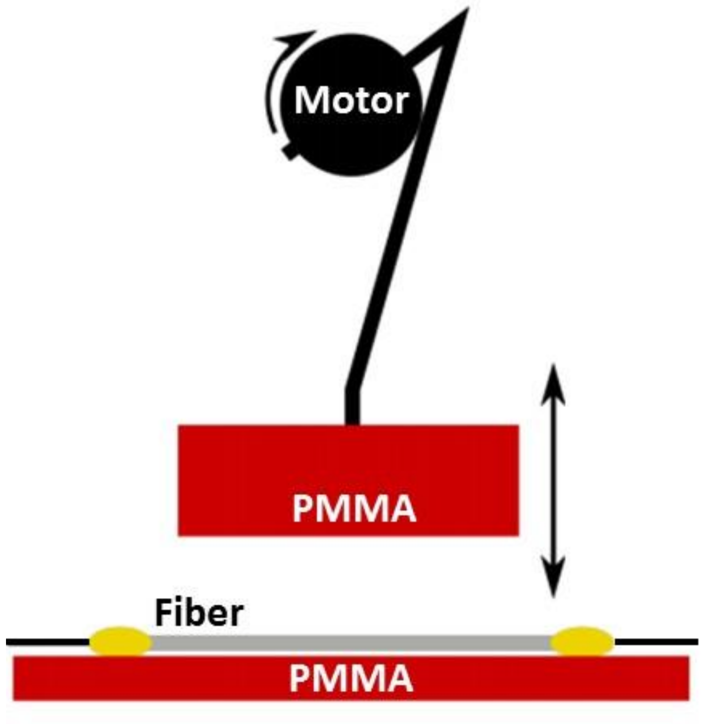

Supplementary Figure 9. Schematic showing the custom fiber compression setup. The length and width of upper PMMA sheet are 30 mm and 14 mm respectively, while the length and diameter of the tested fiber are 40 mm and 0.93 mm respectively. The maximum displacement distance of the PMMA sheet is 16 mm.

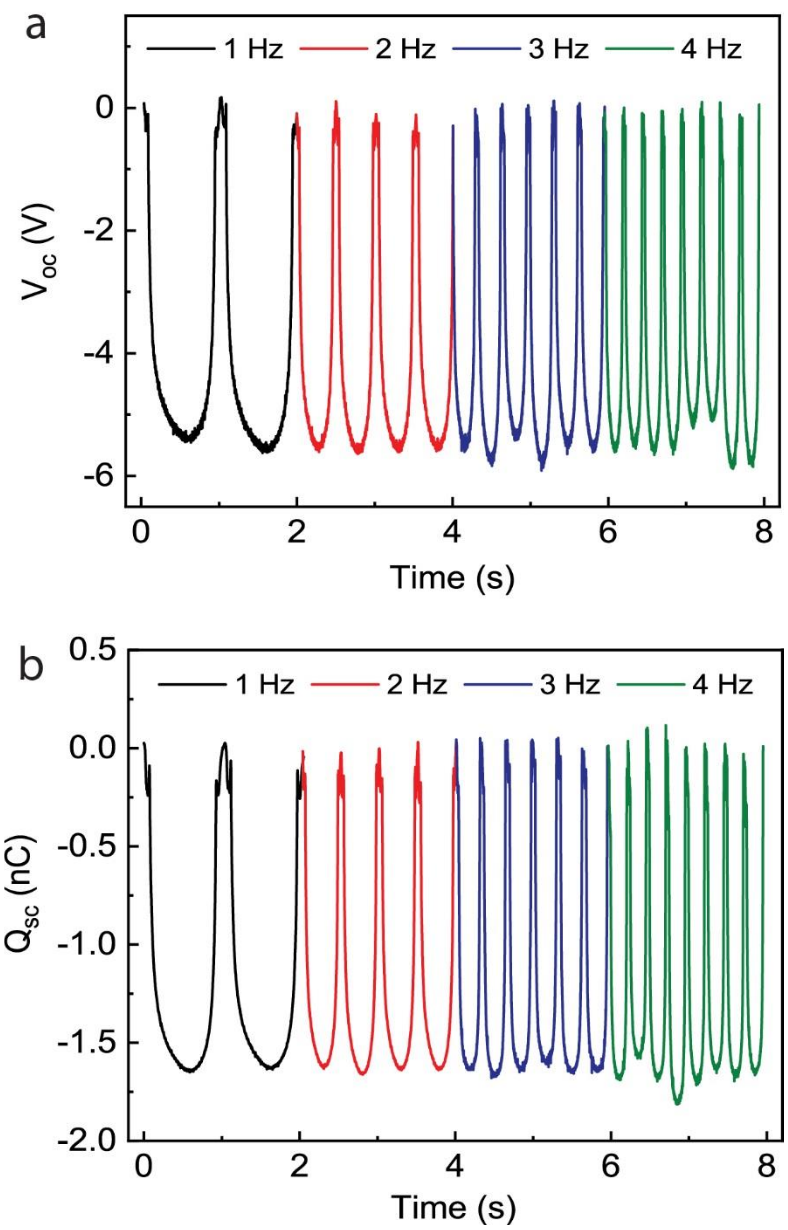

Supplementary Figure 10. Typical electrical output signals by tapping a short fiber. **a** V_{oc} **b** Q_{sc} at various frequencies (effective contact length: 3 cm).

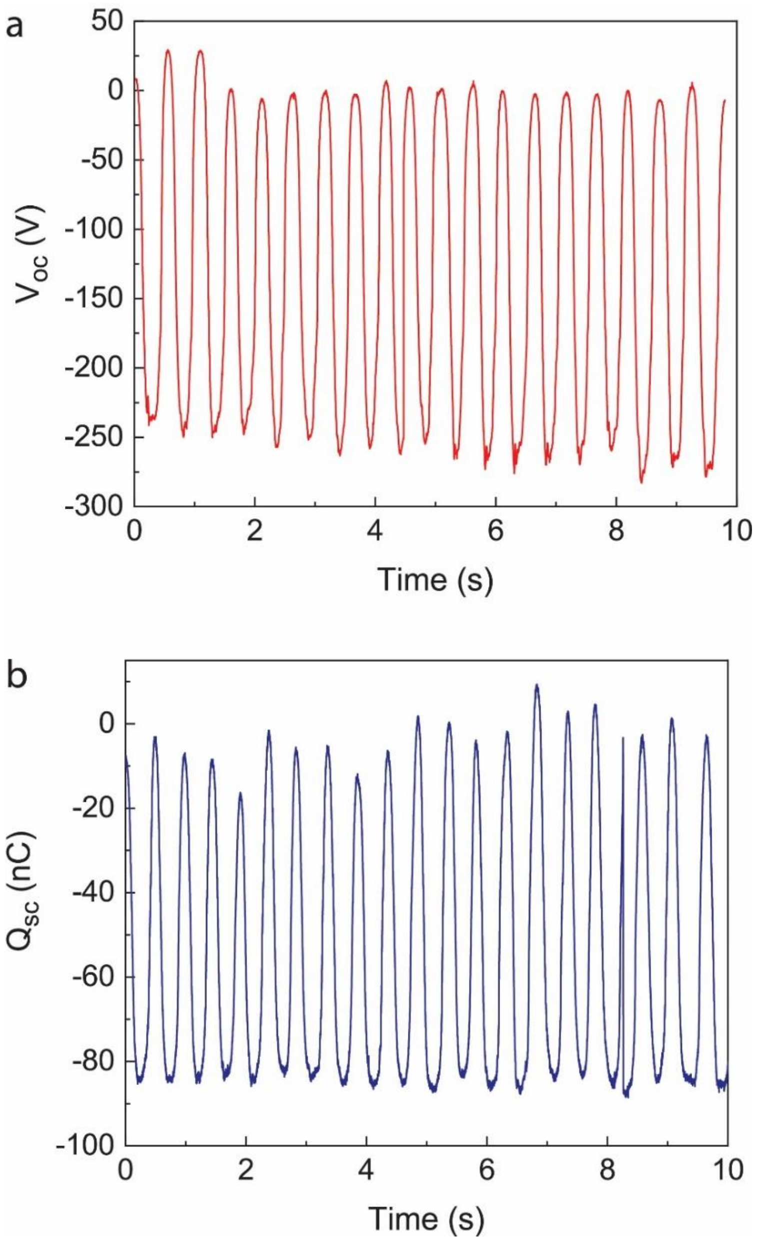

Supplementary Figure 11. Hand-grasping triggered outputs of the TENG textile. a V_{oc} **b** Q_{sc}.

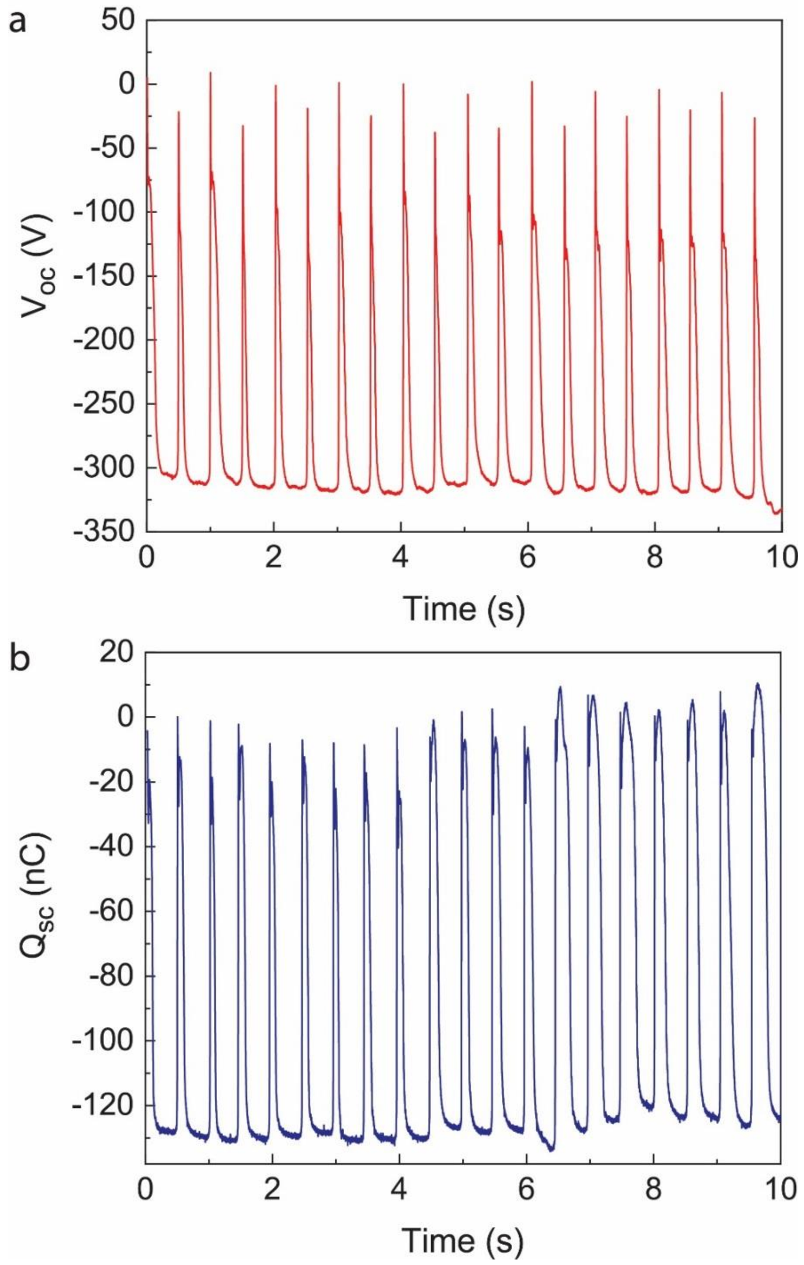

Supplementary Figure 12. Hand-tapping triggered outputs of the TENG textile. a V_{oc} **b** Q_{sc}.

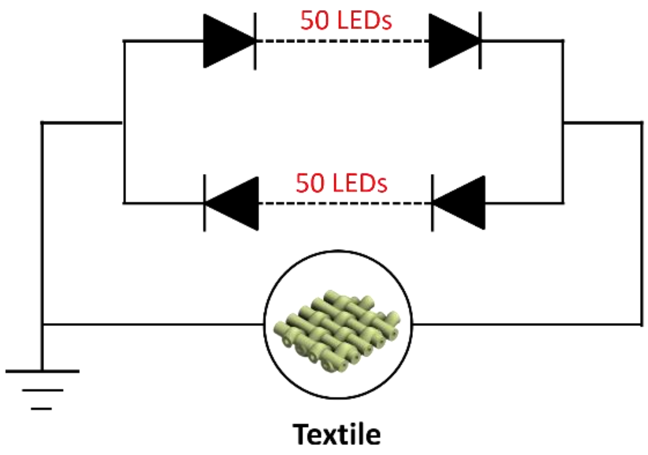

Supplementary Figure 13. Electrical circuit diagram showing the textile as a direct power source to drive 100 LEDs.

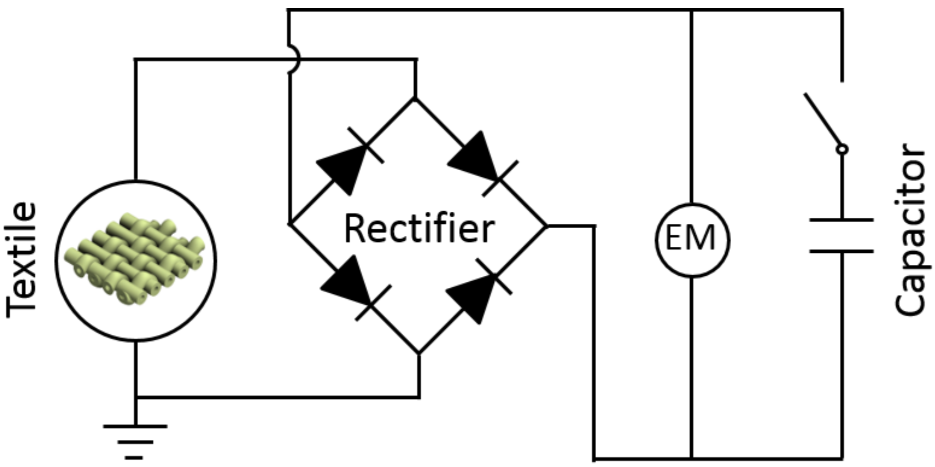

Supplementary Figure 14. Electrical circuit diagram of the textile connected with a bridge rectifier to charge a capacitor.

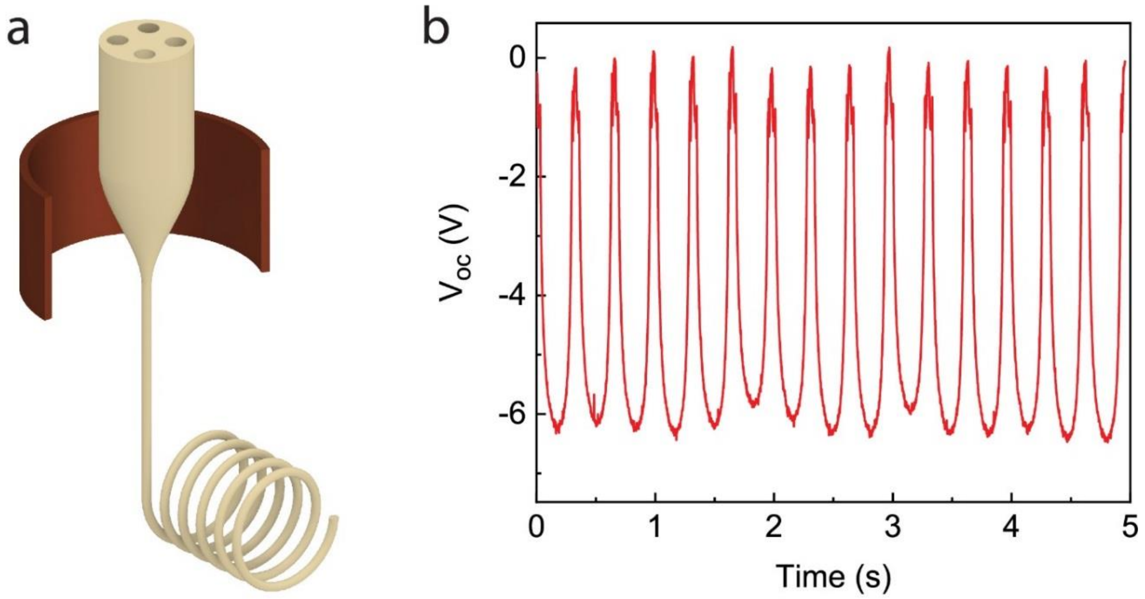

Supplementary Figure 15. TENG fibers with multiple liquid metal electrodes. a The flexibility of fiber design at the preform level enables the scalable fabrication of a complex fiber structure with multiple electrodes. **b** Output performance of such a fiber with the effective contact length of 3 cm.

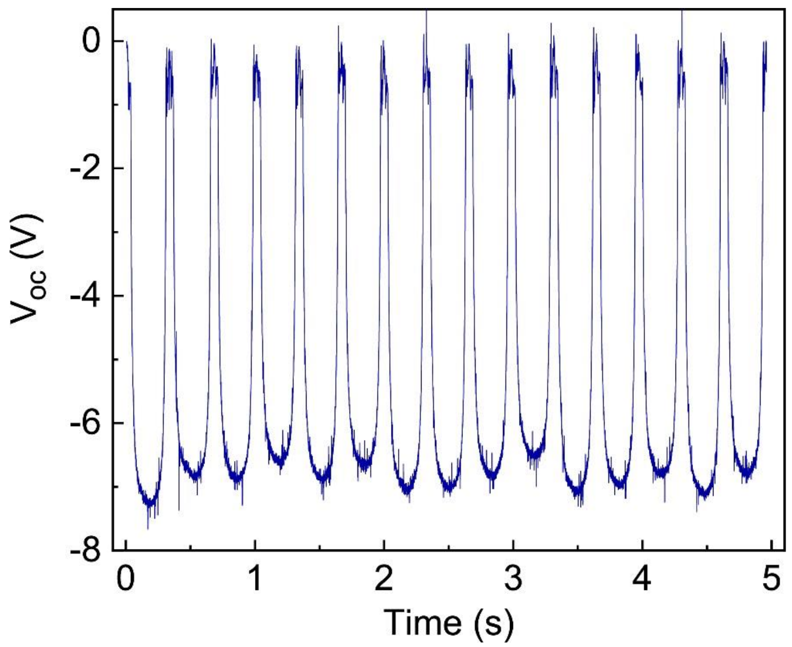

Supplementary Figure 16. Open-circuit voltage of a 3 cm-long micro-textured fiber.

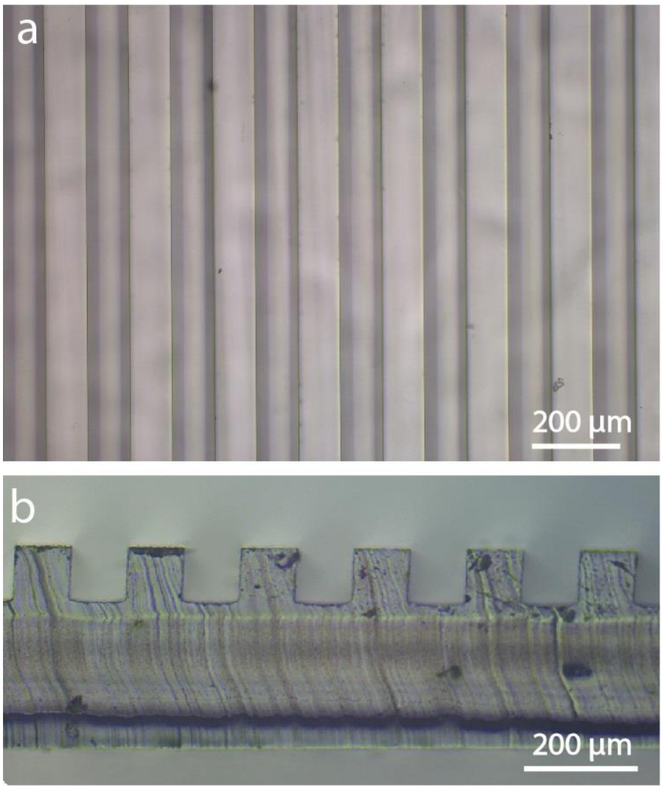

Supplementary Figure 17. Textured Geniomer film for the fabrication of textured preforms.

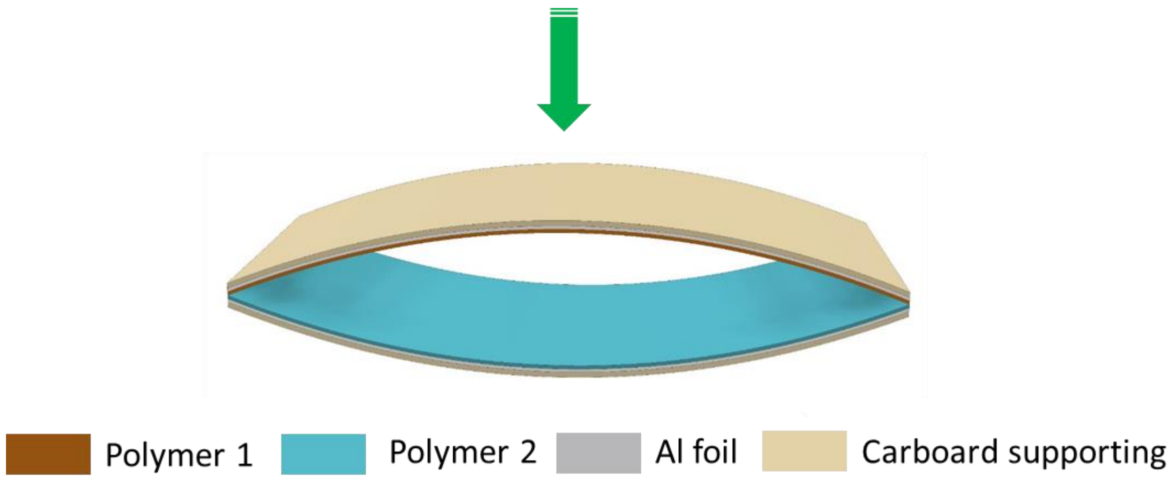

Supplementary Figure 18. Schematic showing 2D planar TENG devices (contact-mode). Such devices are used to determine relative triboelectric polarity of the tested polymers in this work.

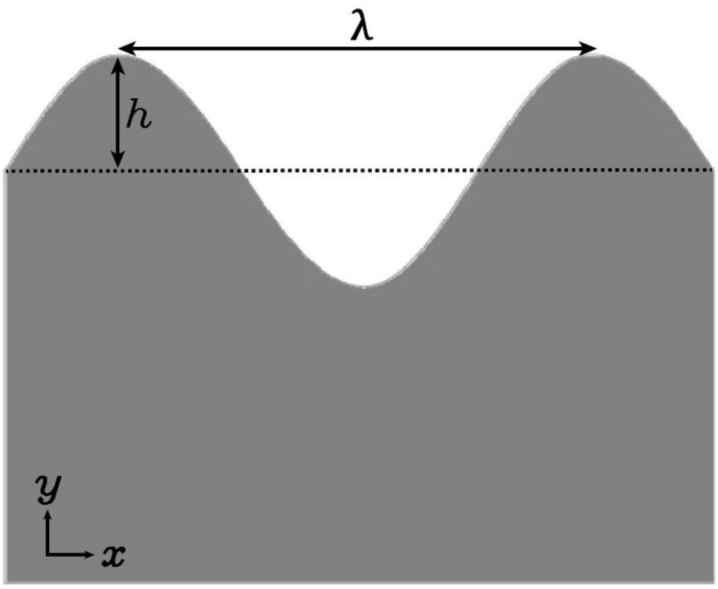

Supplementary Figure 19. Schematic of a sinusoidal surface on a textured fiber with relevant parameters.

Supplementary Tables

Supplementary Table 1. Comparison of the electrical output performance of the triboelectric fiber with other reported fibers.

Fiber length, diameter	Electrode materials	Triboelectric surfaces	Electrical output	Electrical output per volume	Trigger conditions	Stretchability	Reference
3 cm,<br>0.93 mm	Liquid metal	Geniomer//PMMA	5.5 V<br>180 nA (4 Hz)<br>1.6 nC	269.9 V/cm^3<br>8.8 µA/cm^3<br>78.5 nC/cm^3	Compression<br>2 N, 1.6 cm	557%	This work
1.5 cm,<br>1.8 mm	Stainless steel	PDMS//unknown external substrate	4 V<br>0.7 µA (2 Hz)<br>1.25 nC	104.8 V/cm^3<br>18.3 µA/cm^3<br>32.7 nC/cm^3	Compression<br>2.5 cm	« not given »	Advanced Materials 29, 1702648 (2017)[3]
2 cm,<br>6 mm	Stainless steel	Silicone//acrylic	18 V<br>0.3 µA (3 Hz)<br>6 nC	31.8 V/cm^3<br>0.5 µA/cm^3<br>10.6 nC/cm^3	Compression<br>4 cm	« not given »	ACS Nano 11, 9490–9499 (2017)[4]
10 cm,<br>2 mm	Carbon fiber	Silicone//skin	42.9 V<br>0.51 µA (2.5 Hz)	136.6 V/cm^3<br>1.6 µA/cm^3	« not given »	« not given »	ACS Applied Materials and Interfaces 10, 42356–42362 (2018)[5]
11 cm,<br>2.8 mm	Metal wire, Ag NW	Nylon//silicone	4.16 V	6.1 V/cm^3	Stretch 68%	300%	Nano Energy 39, 673–683 (2017)[6]
30 cm,<br>1.8 mm	Helical stainless steel	Silicone//stainless steel	4.2 V	5.5 V/cm^3	Stretch 100%	200%	Nature Communications 10, 1–8 (2019)[7]
5 cm,<br>~2.5 mm	Ag-coated nylon fiber, CNT sheets	Ag-coated Nylon yarn//PVDF-TrFE	24 mV<br>8 nA (10 Hz)<br>10 pC	0.098 V/cm^3<br>0.03 µA/cm^3<br>0.04 nC/cm^3	Stretch 50%	50%	Scientific Reports 6, 35153 (2016)[8]
4 cm,<br>1. 6mm	Ag NW	PTFE//PDMS-Ag NW	10 nA (2 Hz)	0.12 µA/cm^3	Compression<br>0.16 N	50%	Nano Energy 41, 511–518 (2017)[9]
10 cm,<br>4 mm	CNT, coiled Cu wire	Silicone//Cu	140 V<br>75 nA/cm (2 Hz)<br>5 nC/cm	111.4 V/cm^3<br>0.6 µA/cm^3<br>39.8 nC/cm^3	Stretch 50%	100%	Advanced Functional Materials 27, 1604378 (2017)[10]
2.5 cm,<br>~1 mmm	CNT	PMMA//PDMS	1 V<br>150 nA (1 Hz)	50.9 V/cm^3<br>7.6 µA/cm^3	Compression<br>10 N	stretchable	Journal of Materials Chemistry A 5, 6032–6037 (2017)[11]

Supplementary Table 2. Summary of the electrical output performance of the triboelectric textile in this work and other previously reported textiles.

Textile dimension (cm*cm)	Contact area (cm*cm)	Applied force/pressure	Electrode materials	Triboelectric materials	Electrical output	Electrical output per area	*reference*
6*6	6*6	25.5 N<br>7.1 kPa	Liquid metal	Geniomer//Nitrile	490 V<br>175 nC	13.6 V/cm^2<br>4.86 nC/cm^2	This work
10*10	« not given »	« not given »	Ag	Ag//PTFE	23.5 V<br>1.05 µA	0.2 V/cm^2<br>0.01 µA/cm^2	ACS Nano 11, 10733–10741 (2017)[12]
16.5*11.4	« not given »	« not given »	Stainless steel thread	Silicone//skin	200 V<br>200 µA	1.06 V/cm^2<br>1.06 µA/cm^2	Advanced Functional Materials 27, 1604462 (2017)[13]
5*10	5*10	« not given »	Ag fiber fabric	Nylon//polyester	90 V<br>1 µA	1.8 V/cm^2<br>0.02 µA/cm^2	ACS Applied Materials and Interfaces 6, 14695–14701 (2014)[14]
4*4	4*4	11 N<br>6.9 kPa	Stainless steel	Silicone//acrylic	150V<br>2.9 µA (5Hz)<br>52 nC	9.4 V/cm^2<br>0.18 µA/cm^2<br>3.25 nC/cm^2	ACS Nano 11, 9490–9499 (2017)[4]
4.5*8	4.5*8	« not given »	Stainless steel	Polyurethane//polyester	75 V<br>1.2 µA(1Hz)	2.1 V/cm^2<br>0.03 µA/cm^2	ACS Nano 11, 12764–12771 (2017)[15]
4.0*4.5	1.5*1.5	« not given »	Stainless steel	PDMS//unknown external substrate	20 V<br>0.1 µA (5Hz)<br>7 nC<br>(data of one-dielectric-layer)	8.9 V/cm^2<br>0.04 µA/cm^2<br>3.1 nC/cm^2	Advanced Materials 29, 1702648 (2017)[3]
10*10	10*10	« not given »	Ni	Parylene//cotton	40 V<br>5 µA (5Hz)	0.4 V/cm^2<br>0.05 µA/cm^2	Advanced Materials 28, 98–105 (2016)[16]
5*6	5*6	« not given »	Helical structure stainless steel	Silicone//stainless steel	31.8 V	1.06 V/cm^2	Nature Communications 10, 1–8 (2019)[7]
1*1	1*1	9.8 N<br>98 kPa	Al, conductive fabric	Nylon//PVDF	2 V<br>0.2 µA	2 V/cm^2<br>0.2 µA/cm^2	Journal of Materials Chemistry A 6, 22879–22888 (2018)[17]
6*6 (circle)	6*6 (circle)	10 to 20 N<br>3.5 to 7 kPa	Al foil	PVA/Mxene//silk fibroin	118.4 V (10 Hz)	4.2 V/cm^2	Nano Energy 59, 268–276 (2019)[18]
4*4	« not given »	« not given »	Cu	PTFE//Cu	70 V<br>50 µA	4.4 V/cm^2<br>3.12 µA/cm^2	Nature Energy 1, 16138 (2016)[19]
5*5	5*5	10 N<br>4 kPa	PEDOT:PSS coated textile, Al	PEDOT:PSS coated textile//PTFE	49.7 V<br>0.787 µA	2 V/cm^2<br>0.03 µA/cm^2	Nano Energy 57, 338–352 (2019)[20]
4*4	4*4	10 N<br>6.25 kPa	Conductive fabric	PVDF/PTFE//skin	112.7 V<br>1.98 µA	7 V/cm^2<br>0.12 µA/cm^2	Nano Energy 58, 750–758 (2019)[21]
6*4	6*4	« not given »	Cu	Cu//polyimide	4.98 V<br>15.5 mA/m^2	0.2 V/cm^2<br>1.55 µA/cm^2	Advanced Materials 28, 10267–10274 (2016)[22]
5*5	5*5	« not given »	Ni cloth	Parylene//Ni	50 V<br>4 µA	2 V/cm^2<br>0.16 µA/cm^2	Advanced Materials 27, 2472–2478 (2015)[23]
10*10	10*10	1400 N<br>140 kPa	Cu sheet	Cu//PDMS	~195 V	1.95 V/cm^2	Nano Energy 58, 365–374 (2019)[24]
5.3*2.9	5.3*2.9	« not given »	Liquid metal	Silicone	~120 V	7.8 V/cm^2	ACS nano 12, 2027–2034 (2018)[25]
5*5	5*5	300 N<br>120 kPa	Ni	Silicon rubber, Ni//skin	300 V<br>30 µA	12 V/cm^2<br>1.2 µA/cm^2	Nano Energy 39, 562–570 (2017)[26]

3*3	1.5*1.5	« not given »	Au	Water//HCOENP	15 V<br>4 µA	6.7 V/cm^2<br>1.8 $\mu A/cm^2$	Advanced Energy Materials 7, 1–10 (2017)[27]
6*5	6*5	« not given »	Carbon wire	PTFE//carbon wire	~175 V<br>8 µA	5.8 V/cm^2<br>0.27 $\mu A/cm^2$	Nano Energy 50, 536–543 (2018)[28]
5*5	5*5	1 N<br>0.4 kPa	Ag	PET//silicone	45 V	1.8 V/cm^2	Materials Today 32, 84–93 (2019)[29]
7*7	7*7	5 N<br>1 kPa	Ag flake/ PDMS	Skin//HCOENPs/BP/PET	350 V	7.1 V/cm^2<br>0.65 $\mu A/cm^2$ (2 Hz)	Nature Communications 9, 1–9 (2018)[30]
9*10	9*10	« not given »	Ag-coated fabric	PTFE//cotton yarn/Ag (free-standing mode)	900 V<br>19 µA	10 V/cm^2<br>0.21 $\mu A/cm^2$	Nano Energy 58, 375–383 (2019)[31]

Note: The calculation of electrical contact per area is calculated based on the contact area provided it is known. As for the devices with unknown contact area, the calculation is based on the textile dimension.

Supplementary Note 1

Discussion on the surface area enhancement of micro-textured fibers

Compared with its original shape at the preform level (Supplementary Figure 17), the periodic texture was well maintained on the fiber, despite slight structural smoothing. Based on the cross-section of the fiber, it is reasonable to approximate that the texture can be expressed by a sinusoidal function $y\ (x) = hsin(\frac{2\pi}{\lambda}x)$, where h and λ are the amplitude and period of the perturbation respectively (Supplementary Figure 19). According to the original dimensional design in the preform, h is estimated to be $\frac{\lambda}{4}$. We compare the surface area of a sinusoidal patterned fiber with a smooth surface fiber in the range of one period, under the assumption that they have the same fiber length and cross-sectional diameter. We assume that the surface area enhancement is represented by $\frac{s-\lambda}{\lambda}$, where s is the arc length of the sine function over one period λ, which equals to $\int_0^{\lambda}\sqrt{1+y'^2(x)}dx$. The calculated surface area enhancement is 46%, which results in outputs with similar enhancement if the triboelectric surfaces are in full contact under external stimuli.

Supplementary References

1. Diaz, A. F. & Felix-Navarro, R. M. A semi-quantitative tribo-electric series for polymeric materials: the influence of chemical structure and properties. *J. Electrostat.* **62**, 277–290 (2004).

2. Wang, Z. L. Triboelectric Nanogenerators as New Energy Technology for Self-Powered Systems and as Active Mechanical and Chemical Sensors. *ACS Nano* **7**, 9533–9557 (2013).

3. Dong, K. *et al.* 3D Orthogonal Woven Triboelectric Nanogenerator for Effective Biomechanical Energy Harvesting and as Self-Powered Active Motion Sensors. *Adv. Mater.* **29**, 1702648 (2017).

4. Dong, K. *et al.* A Highly Stretchable and Washable All-Yarn-Based Self-Charging Knitting Power Textile Composed of Fiber Triboelectric Nanogenerators and Supercapacitors. *ACS Nano* **11**, 9490–9499 (2017).

5. Yang, Y. *et al.* Coaxial Triboelectric Nanogenerator and Supercapacitor Fiber-Based Self-Charging Power Fabric. *ACS Appl. Mater. Interfaces* **10**, 42356–42362 (2018).

6. Gong, W. *et al.* A wearable, fibroid, self-powered active kinematic sensor based on stretchable sheath-core structural triboelectric fibers. *Nano Energy* **39**, 673–683 (2017).

7. Gong, W. *et al.* Continuous and scalable manufacture of amphibious energy yarns and textiles. *Nat. Commun.* **10**, 1–8 (2019).

8. Sim, H. J. *et al.* Stretchable Triboelectric Fiber for Self-powered Kinematic Sensing Textile. *Sci. Rep.* **6**, 35153 (2016).

9. Cheng, Y. *et al.* A stretchable fiber nanogenerator for versatile mechanical energy harvesting and self-powered full-range personal healthcare monitoring. *Nano Energy* **41**, 511–518 (2017).

10. He, X. *et al.* A Highly Stretchable Fiber-Based Triboelectric Nanogenerator for Self-Powered Wearable Electronics. *Adv. Funct. Mater.* **27**, 1604378 (2017).

11. Yu, X. *et al.* A coaxial triboelectric nanogenerator fiber for energy harvesting and sensing under deformation. *J. Mater. Chem. A* **5**, 6032–6037 (2017).

12. Kwak, S. S. *et al.* Fully Stretchable Textile Triboelectric Nanogenerator with Knitted Fabric Structures. *ACS Nano* **11**, 10733–10741 (2017).

13. Lai, Y.-C. *et al.* Single-Thread-Based Wearable and Highly Stretchable Triboelectric Nanogenerators and Their Applications in Cloth-Based Self-Powered Human-Interactive and Biomedical Sensing. *Adv. Funct. Mater.* **27**, 1604462 (2017).

14. Zhou, T. *et al.* Woven structured triboelectric nanogenerator for wearable devices. *ACS Appl. Mater. Interfaces* **6**, 14695–14701 (2014).

15. Yu, A. *et al.* Core-Shell-Yarn-Based Triboelectric Nanogenerator Textiles as Power Cloths. *ACS Nano* **11**, 12764–12771 (2017).

16. Pu, X. *et al.* Wearable Self-Charging Power Textile Based on Flexible Yarn Supercapacitors and Fabric Nanogenerators. *Adv. Mater.* **28**, 98–105 (2016).

17. Shin, Y. E. *et al.* Sewing machine stitching of polyvinylidene fluoride fibers: Programmable textile patterns for wearable triboelectric sensors. *J. Mater. Chem. A* **6**, 22879–22888 (2018).

18. Jiang, C. *et al.* All-electrospun flexible triboelectric nanogenerator based on metallic MXene nanosheets. *Nano Energy* **59**, 268–276 (2019).

19. Chen, J. *et al.* Micro-cable structured textile for simultaneously harvesting solar and mechanical energy. *Nat. Energy* **1**, 16138 (2016).

20. He, T. *et al.* Beyond energy harvesting - multi-functional triboelectric nanosensors on a textile. *Nano Energy* **57**, 338–352 (2019).

21. Qiu, Q. *et al.* Highly flexible, breathable, tailorable and washable power generation fabrics for wearable electronics. *Nano Energy* **58**, 750–758 (2019).

22. Zhao, Z. *et al.* Machine-Washable Textile Triboelectric Nanogenerators for Effective Human Respiratory Monitoring through Loom Weaving of Metallic Yarns. *Adv. Mater.* **28**, 10267–10274 (2016).

23. Pu, X. *et al.* A self-charging power unit by integration of a textile triboelectric nanogenerator and a flexible lithium-ion battery for wearable electronics. *Adv. Mater.* **27**, 2472–2478 (2015).

24. Gong, J. *et al.* Towards truly wearable energy harvesters with full structural integrity of fiber materials. *Nano Energy* **58**, 365–374 (2019).

25. Yang, Y. *et al.* Liquid-metal-based super-stretchable and structure-designable triboelectric nanogenerator for wearable electronics. *ACS nano* **12**, 2027–2034 (2018).

26. Tian, Z. *et al.* Performance-boosted triboelectric textile for harvesting human motion energy. *Nano Energy* **39**, 562–570 (2017).

27. Xiong, J. *et al.* Wearable All-Fabric-Based Triboelectric Generator for Water Energy Harvesting. *Adv. Energy Mater.* **7**, 1–10 (2017).

28. Chen, J. *et al.* Traditional weaving craft for one-piece self-charging power textile for wearable electronics. *Nano Energy* **50**, 536–543 (2018).

29. Chen, C. *et al.* 3D double-faced interlock fabric triboelectric nanogenerator for bio-motion energy harvesting and as self-powered stretching and 3D tactile sensors. *Mater. Today* **32**, 84–93 (2019).

30. Xiong, J. *et al.* Skin-touch-actuated textile-based triboelectric nanogenerator with black phosphorus for durable biomechanical energy harvesting. *Nat. Commun.* **9**, 1–9 (2018).

31. Huang, T. *et al.* Fabric texture design for boosting the performance of a knitted washable textile triboelectric nanogenerator as wearable power. *Nano Energy* **58**, 375–383 (2019).
