# Supplementary material for: High-efficiency super-elastic liquid metal based triboelectric fibers and textiles
Source: Nat Commun. 2020 Jul 15;11:3537. doi: 10.1038/s41467-020-17345-8 (PMC7363815; doi:10.1038/s41467-020-17345-8)
Supplement: Supplementary file 2 — Description of Additional Supplementary Files [file 41467_2020_17345_MOESM2_ESM.pdf]

Description of Additional Supplementary Files

File Name: Supplementary Movie 1
Description: A triboelectric textile that powers 100 LEDs.
